# Editorial: Clinical guidelines in bipolar disorder: applications and evaluation

**DOI:** 10.3389/fpsyt.2025.1636373

**Published:** 2025-06-11

**Authors:** Heinz Grunze

**Affiliations:** ^1^ Department of Psychiatry, Psychiatrie Schwäbisch Hall, Ringstraße, Germany; ^2^ Department of Psychiatry, Paracelsus Medical University, Nuremberg, Germany

**Keywords:** bipolar disorder, psychoeducation, mindfulness, guideline, bipolar depression

The call for this Research Topic was well received, and altogether 16 manuscripts have been submitted, out of which 7 were accepted after peer review. Areas covered are the challenges in compiling up-to-date bipolar guidelines, their application in everyday practice, new diagnostic approaches and biological markers both for diagnosis and treatment outcomes, and finally the interplay between somatic disease and bipolar disorder.

The scholarly paper by Fountoulakis et al. details the challenges when compiling a bipolar guideline, including methodological traps, the complexity and uniqueness of the clinical picture and the therapeutical options available, special issues including gender, pregnancy, and the different views of therapists and patients. The lead author is a well experienced and respected authority in the development of guidelines. He notes that in the literature, instead of a holistic view, bipolar disorder is mostly treated as a fragmented condition with each fragment studied separately. Thus, the development of a comprehensive single algorithm for the continuous treatment of BD becomes extremely difficult, demanding an exhaustive review of the existing literature, searching for unpublished data and digging deep into them to comprehend their nature. Isolated aspects of the disorder need to be synthesized into a comprehensive network of decision-making that will incorporate the knowledge of the past with decisions for the present by having the mind in the future.

It appears to be common sense throughout guidelines to try and avoid polypharmacy and to use medication proven effective and recommended in bipolar disorder. Analyzing data from the ongoing Bavarian multicenter drug safety project Pharmaco-Epidemiology and Vigilance (Pharmako-EpiVig), Kriner et al. found that clinical reality does not reflect guideline recommendations, at least in bipolar depression. More than one-third of patients were not prescribed any drug explicitly recommended for treatment of BPD, and only 6% of the patients received monotherapy with a recommended medication, whereas more than one-third of patients were administered ≥4 psychotropic drugs simultaneously. They also observed a trend towards prescribing more lithium instead of valproate and an increasing preference towards atypical antipsychotics, and, in summary, a remarkable heterogeneity in treatment regimens.

Not only pharmaceutical treatment, but also the application of psychoeducation shows remarkable heterogeneity, possibly reflecting the complexity of the disorder. The review by Levrat et al. summarizes the literature on current practices and forms of psychoeducation in the management of patients with bipolar disorder including only randomized controlled trials. The literature search yielded 381 studies of which seventy articles were finally included. Different forms of psychoeducation were compared on its own or combined with other psychosocial interventions. In summary, psychoeducation appears important in the treatment of BD, as it leads to a decrease in relapses, mood episodes, hospitalizations, and improved functioning or quality of life. In addition, some forms of psychoeducation were able to increase patient’s level of knowledge of pharmacological treatment and the disorder or compliance with medication, as well as reduced self-stigma.

Compared to psychoeducation, Mindfulness Based Cognitive Therapy (MBCT) has been studies less extensively in Bipolar disorder so far. Carracedo-Sanchidrián et al. report on their randomized controlled trial testing the effect of adjunctive mindfulness-based cognitive therapy versus psychoeducational intervention on plasma brain-derived neurotrophic factor (BDNF) and cognitive function in bipolar patients. Of special interest, this trial combined and related psychometric outcomes with a biological measure. The hypothesis was that MBCT would improve cognitive functioning and BDNF more than psychoeducation and treatment as usual (TAU). Eighty-four bipolar outpatients were recruited and assessed at baseline, 8 weeks and 6 months. The result was negative, with MBCT not achieving better results than Psychoeducation or TAU. The authors suggest that the negative outcome might be a result of the fact that TAU is already quite effective in mildly and moderately ill outpatients, so that effectiveness can hardly been topped by additional psychotherapies.

Two articles of this Research Topic focussed on biological markers of bipolar disorder. For the editors of this Research Topic, it was quite surprising that the old-fashioned EEG can still serve as a valuable tool supporting a bipolar depression diagnosis. The study by Yang et al. systematically evaluate the efficacy of the three classic EEG paradigms- eyes open, eyes closed, and free viewing-in diagnosing bipolar disorder. They compared EEGs from 28 individuals diagnosed with BD and 42 healthy controls. The eyes closed paradigm turned out to be a superior, straightforward EEG experimental approach for the diagnosis of bipolar depression.

However, when it comes to diagnostic tools, nowadays research is focusing more on genes that may regulate neurodegenerative processes such as oxidative stress. Wu et al. identified three hub genes and crucial pathways linked to oxidative stress in bipolar depression using bioinformatics analysis. These three potential biomarkers for bipolar disorder were involved in neuronal signal transduction, oxidative phosphorylation, and metabolic obstacle pathways, and may become a future target for diagnosing and treating bipolar disorder.

Finally, Kong et al. examined the relationship between depression and hepatobiliary diseases using genome-wide association studies. They looked into a potential bidirectional causal relationship between depression and various hepatobiliary diseases, and found that depression is a susceptibility factor for non-alcoholic fatty liver disease, with the causal effect of genetic susceptibility to depression on non-alcoholic fatty liver disease being mediated by waist-hip ratio, hypertension, and daytime nap. Although this study did not look specifically into bipolar depression, the mediating factors are also abundant in bipolar depressed patients, and may explain the increased incidence of hepatic disease also in bipolar disorder (1).

In summary, this compilation depicts different aspects of diagnosing and treating bipolar disorder, but also the difficulties to extract a guideline fitting a majority of patients. Bipolar disorder remains a complex disorder demanding a high degree of personalized medicine.
